# Anti-Aging in *Caenorhabditis elegans* of Polysaccharides from *Polygonatum cyrtonema* Hua

**DOI:** 10.3390/molecules29061276

**Published:** 2024-03-13

**Authors:** Xue Zhang, Qi Chen, Linzhen Chen, Xiaolu Chen, Zhiqiang Ma

**Affiliations:** Beijing Key Laboratory for Quality Evaluation of Chinese Materia Medica, School of Chinese Materia Medica, Beijing University of Chinese Medicine, Beijing 102488, China; zhangxuezyhx@163.com (X.Z.); chen_qi0823@163.com (Q.C.); chenlinzhen123@163.com (L.C.); chenxiaolu0109@163.com (X.C.)

**Keywords:** *Polygonatum cyrtonema* Hua, polysaccharides, *Caenorhabditis elegans*, separation and purification, anti-aging

## Abstract

*Polygonatum cyrtonema* Hua, the dried rhizome of *Polygonum multiflorum* from the Liliaceae family, is a widely used medicinal herb with a long history of application. Its main active ingredients are polysaccharides, which have been demonstrated in contemporary studies to effectively delay the aging process. In the present study, homogeneous polysaccharide (PCP-1) was obtained after the purification and isolation of polysaccharides from *Polygonatum cyrtonema* Hua (PCP). The anti-aging activities of both were compared, and the possible mechanism of action for exerting anti-aging activity was explored using *Caenorhabditis elegans* (*C. elegans*). Research has indicated that PCP and PCP-1 exhibit potent anti-oxidant and anti-aging properties. Of particular note is that PCP-1 acts better than PCP. The two were able to prolong the lifespan of nematodes, improve the stress resistance of nematodes, reduce the accumulation of lipofuscin in the intestine, decrease the content of ROS and MDA in the body, increase the activity of the antioxidant enzymes SOD and CAT, promote the nuclear translocation of DAF-16, down-regulate the mRNA levels of the *age-1* and *daf-2* genes of the IIS pathway in nematodes, and up-regulate the expression of the *daf-16*, *skn-1*, *sod-3*, and *hsp-16.2* genes. Based on the aforementioned findings, it is possible that the mechanism by which PCP and PCP-1 exert anti-aging effects may be through negative regulation of the IIS pathway, activation of the transcription factor DAF-16/FOXO, and enhancement of oxidative defenses and stress resistance in nematodes. Overall, the present study illustrated the great potential of polysaccharides from *Polygonatum cyrtonema* Hua in anti-aging and antioxidant activities. Specifically, PCP-1 demonstrated superior characteristics, which provides a reference for the future development of *Polygonatum cyrtonema* Hua polysaccharides.

## 1. Introduction

Aging is a spontaneous and inevitable process that occurs in organisms over time. It is a complex biological phenomenon that manifests itself in the form of degenerative structural changes and functional deterioration and the weakening of adaptability and resistance [1]. With age, the metabolic rate of the organism slows down, organ function declines, and the incidence of various diseases increases [2]. The aging of the population in the current society is increasing, which is a great challenge to the healthcare industry and increases socio-economic burden. On the other hand, the market for anti-aging products has been gradually expanding in recent years due to the global trend toward balanced diets and healthy eating. There are younger people in the population in addition to middle-aged and elderly individuals. As the concept of naturalness and wellness has taken hold in the minds of the people, developers have been searching for natural plant ingredients with anti-aging properties to meet general public demands and address health concerns. Therefore, research on the aging process and anti-aging natural medicines is extremely important.

The advantages of *Caenorhabditis elegans* (*C. elegans*) include its short life cycle; ease of manipulation; ability to mimic natural aging; small size; rapid reproduction; and simple, well-developed reproductive and nervous systems, making them ideal biological models for conducting studies on aging [3,4]. *C. elegans*, favored by many researchers, is highly conserved with 60% to 80% homology with human genes [5]. Many researchers are now using *C. elegans* to evaluate the anti-aging activity of drugs and to conduct mechanistic studies.

Polysaccharides, as one of the active ingredients in traditional Chinese medicine, are safe, non-toxic, and rich in biological activity. Polysaccharides have a wide range of beneficial pharmacological effects, including strong immune-regulating, antioxidant, anti-aging, hypoglycemic, and anti-tumor properties [6]. Among them, polysaccharides are more effective at delaying the aging process and work through multiple pathways to have anti-aging benefits. For instance, the insulin/insulin-like growth factor signaling pathway (IIS pathway) is by far the most exhaustive signaling pathway involved in lifespan regulation. This pathway can be involved in regulating the lifespan of a wide range of organisms, including humans and *C. elegans* [7]. Modern research has demonstrated the ability of several herbal polysaccharide components to slow down the aging process. Neutral polysaccharides from *Rehmannia glutinosa* were found to be a functional pharmaceutical ingredient to increase stress resistance and extend the life of *C. elegans* via the IIS [8]. The other finding [9] showed that *Angelica sinensis* polysaccharides remarkably extended lifespan, increased reproduction, improved climbing ability, and increased resistance to starvation and oxidative stress in aged flies, mainly via inhibiting IIS and TOR signaling and boosting antioxidant ability. Using the mouse as a model organism, Li et al. demonstrated that the *Hizikia fusiforme* polyphenol–polysaccharide complex has potential in the development of natural anti-aging drugs [10]. Polysaccharides from various natural plants have been shown to have anti-aging effects in a wide range of model organisms, leading to a growing interest in polysaccharides.

In the research presented here, a homogeneous polysaccharide (PCP-1) was isolated from the crude polysaccharides of *Polygonatum cyrtonema* Hua (PCP), and its preliminary characterized structure was analyzed. Subsequently, using *C. elegans* as a model organism, we compared the anti-aging activities of PCP and PCP-1 with multiple indicators (such as nematode lifespan, stress resistance, lipofuscin, etc.) and explored their mechanisms of action. This research is thus dedicated to exploring the immense possibilities of *Polygonatum cyrtonema* Hua polysaccharides in the field of anti-aging.

## 2. Results

### 2.1. UV Spectroscopy of PCP-1

**Figure 1:**
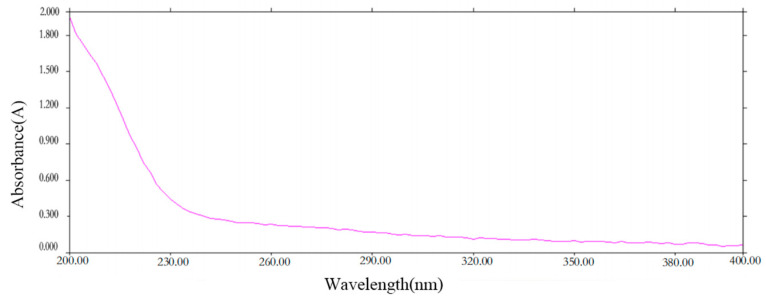
Ultraviolet spectrum of PCP-1.

The UV spectrum scanning graph (Figure 1) showed that PCP-1 has no UV absorption at 260 nm and 280 nm. This is an indication that PCP-1 is free of nucleic acids and proteins.

### 2.2. Monosaccharide Composition of PCP-1

**Figure 2:**
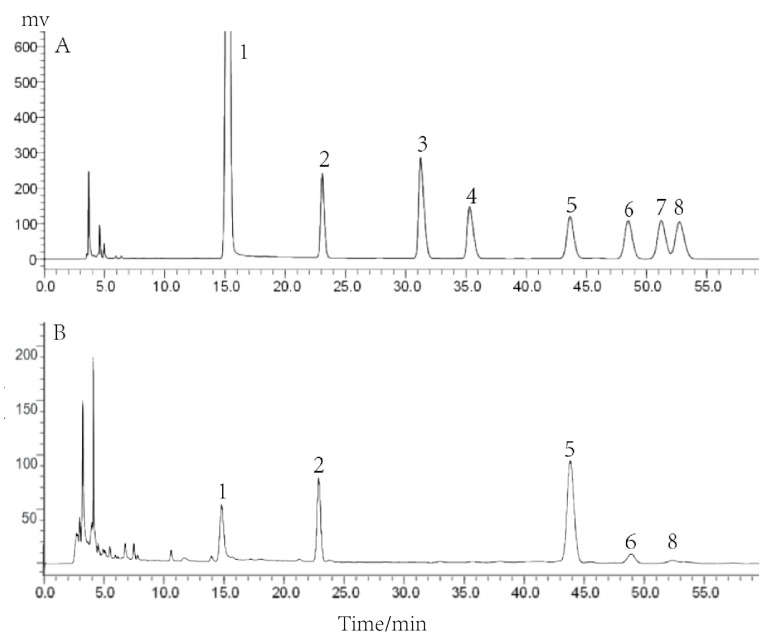
(**A**) HPLC analysis of mixed monosaccharide standards; (**B**) HPLC analysis of the monosaccharide composition of PCP-1. Numbers 1–8, respectively, refer to PMP, mannose, rhamnose, galacturonic acid, glucose, galactose, xylose, and arabinose.

In our previous experiments, we determined the purity and relative molecular mass of PCP-1 and analyzed it by means of FT-IR spectroscopy. The results showed that it had high purity, and its relative molecular mass was 3350 Da. Based on this, PCP-1 was analyzed for its monosaccharide compositions in the research. PCP-1 is mainly composed of four monosaccharides: mannose, glucose, galactose, and arabinose, of which glucose accounts for the largest share (Figure 2). By the standard external method, the molar ratio of the above four monosaccharides was calculated to be 1:2.67:0.25:0.089.

### 2.3. Lifespan Analysis

**Figure 3:**
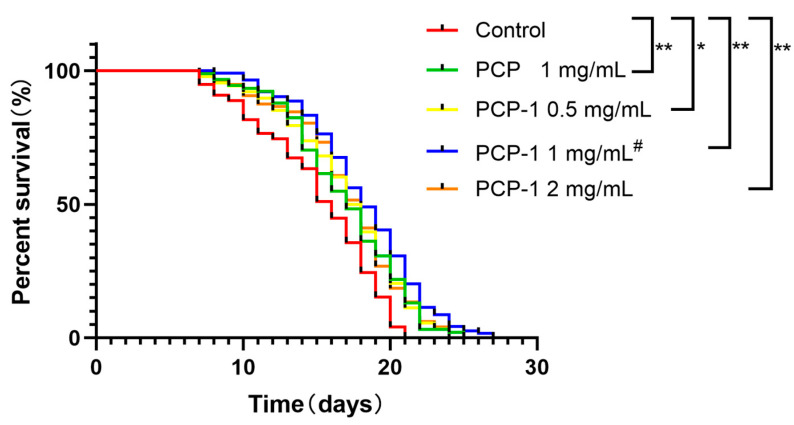
Effect of polysaccharides on nematode longevity. * and ** indicate the different concentrations of administered groups vs. the control group. # indicate PCP vs. PCP-1 (1 mg/mL).

Longevity is the preferred indicator of a drug’s ability to slow senescence and improve the physiological status of *C. elegans*. The antiaging activity of polysaccharides from *Polygonatum cyrtonema* Hua was evaluated by assaying the lifespan expectancy of *C. elegans* in different administered groups and the control group (Table 1). The survival curves for all groups administered at various concentrations were shifted to the right relative to the control group (Figure 3). The maximum increase in worm lifespan was recorded when the concentration of PCP-1 was 1 mg/mL, indicating that the anti-aging effect of PCP-1 was superior to that of PCP at the equivalent concentration.

### 2.4. Effect of Polysaccharides on Stress Resistance in Nematodes

**Figure 4:**
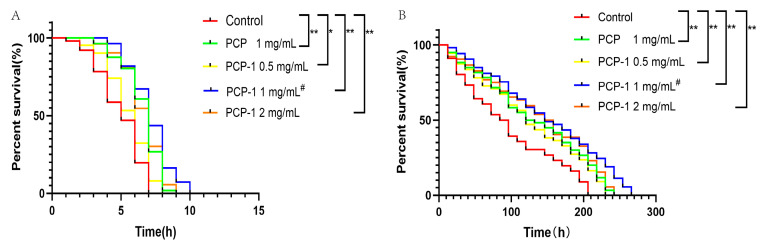
(**A**) Effects of PCP and PCP-1 on the survival rate of *C. elegans* during thermal stress. (**B**) Effects of PCP and PCP-1 on the survival rate of *C. elegans* during oxidative stress. * and ** indicate the different concentrations of administered groups vs. the control group. # indicate PCP vs. PCP-1 (1 mg/mL).

The resistance of nematodes to acute stress is closely correlated with the mechanism of lifespan extension. The greater the tolerance to external stimulation, the longer the lifespan [11]. Stimulation in high-temperature environments leads to oxidative damage in the body and disruption of protein homeostasis. A nematode body under environmental stimulation with a heat shock response will rapidly encode heat shock protein (HSP) to increase its tolerance to high temperatures [12]. The results, as illustrated in Figure 4, reveal that compared with the control group, different concentrations of drug groups shifted the survival curve of nematodes to the right and enhanced their heat resistance under the heat stress environment, with statistically significant differences in each administration group.

Redox homeostasis is regulated by the balance of pro-oxidants and antioxidants. When the homeostatic balance is disrupted, the organism will produce excessive reactive oxygen radicals, which will damage cellular lipids, proteins, DNA, etc. This will affect the physiological functions of cells, leading to organismal tissue damage and aging. Therefore, oxidative stress is a driver of age-related diseases and aging [13,14]. In oxidative stress assays, strong oxidants such as H_2_O_2_, walnut quinone, or paraquat are commonly used to model oxidative stress in nematodes [15,16]. Plant polysaccharide components have been widely reported to extend nematode lifespans by resisting oxidative stress. Polysaccharides can not only increase the activities of the antioxidant enzymes SOD, CAT, and GSH-PX but can also regulate the expression of related antioxidant genes in nematodes to resist oxidative stress [17]. In this article, paraquat was used to induce oxidative stress in nematodes to assess the antioxidant capacity associated with nematodes after treatment with polysaccharides. Under oxidative stress conditions, the survival curves of the nematodes were shifted to the right by the different exposure groups compared to the control group, with statistically significant differences (*p* < 0.01) in all dosing groups. The anti-stress data demonstrated that PCP and PCP-1 had better antioxidant effects. They were able to improve the resistance of nematodes to oxidative stress and heat stress, which in turn prolonged the lifespan of *C. elegans*.

### 2.5. Effect of Polysaccharides on the Reproductive Capacity of Nematodes

**Figure 5:**
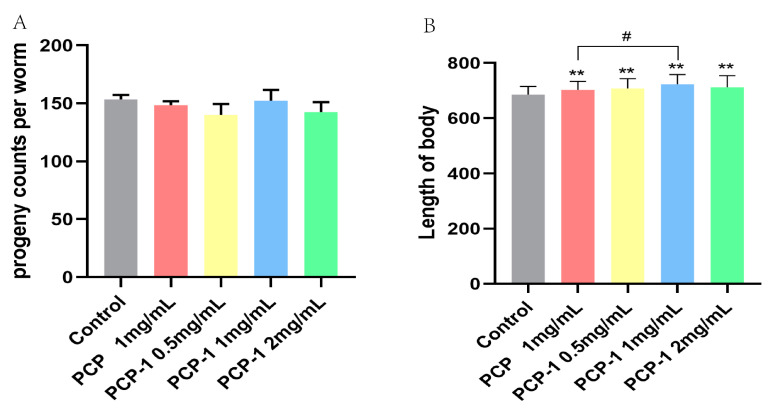
(**A**) Influence of PCP and PCP-1 on the progeny of *C. elegans*. (**B**) Influence of PCP and PCP-1 on the length of *C. elegans*. ** indicate the different concentrations of administered groups vs. the control group. # indicate PCP vs. PCP-1 (1 mg/mL).

Nematode fecundity and body length are essential indicators of whether a medicine will affect nematode body functions. After three days of PCP and PCP-1 administration, there was no significant difference (Figure 5) in the total number of nematode offspring in each administration group versus the control group (*p* > 0.05). The body length showed highly significant statistical differences (*p* < 0.01) between the PCP and varying levels of PCP-1 groups compared to the control group (Figure 5). Scholarly research [18] revealed that the removal of nematode germ cells prolonged the nematodes’ lifespan. In other words, it is a “trade-off mechanism” between fertility and longevity [19]; that is, the substance prolongs longevity at the expense of fertility [20]. In this work, we have observed that PCP and PCP-1 were not reproductively toxic, proposing that the above test drugs prolong nematode lifespan not through a “trade-off” mechanism. At the same time, PCP and PCP-1 can also promote the growth and development of nematodes in body-length experiments, which suggests two of the above polysaccharides do not have biotoxic effects on nematodes.

### 2.6. Reduction of Lipofuscin Accumulation by Polysaccharides in C. elegans

**Figure 6:**
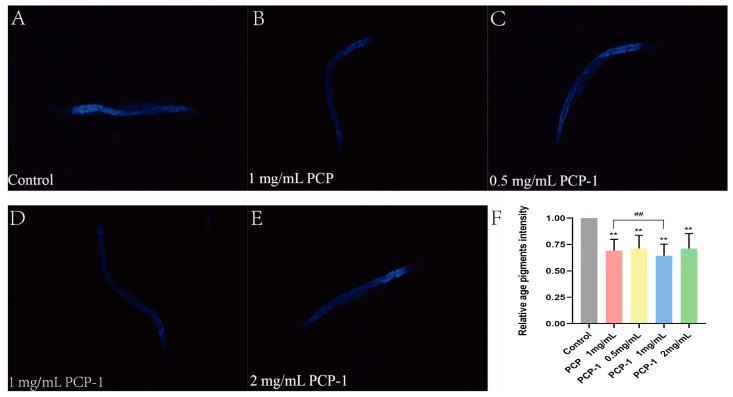
(**A**–**E**) Blue fluorescence produced by lipofuscin in nematodes of each group under fluorescent irradiation. (**F**) Effects of PCP and PCP-1 on lipofuscin in nematodes. ** indicate the different concentrations of administered groups vs. the control group. ## indicate PCP vs. PCP-1 (1 mg/mL).

In order to investigate the effect of polysaccharides from *Polygonatum cyrtonema* Hua on the senescence status of nematodes, lipofuscin accumulation experiments were carried out in this trial. Lipofuscin is an effective biomarker for evaluating the level of organismal aging [21]. With age, lipofuscin is deposited in greater amounts in the cells of the organism. This leads to a slowing down of cellular metabolism and a decrease in activity, resulting in a decline in organ function. These facts contribute to the aging of the organism [22]. Under a fluorescence microscope, lipofuscin in nematodes can be observed to show spontaneous blue fluorescence. The fluorescence accumulation level of lipofuscin in nematodes was reduced (Figure 6) in all dosing groups compared to the control group, and the results were highly significant (*p* < 0.01). Among them, when the concentration of PCP-1 was 1 mg/mL, the level of lipofuscin was reduced by 35.64% relative to the control group.

### 2.7. ABTS and DPPH Radical Scavenging Activity of PCP and PCP-1

**Figure 7:**
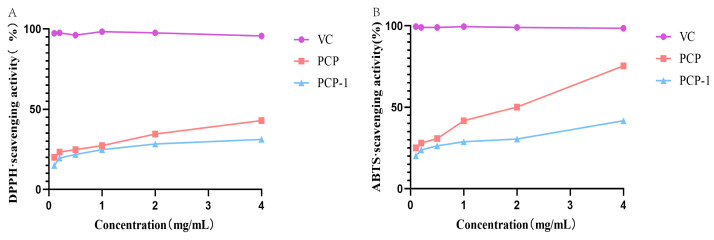
(**A**) DPPH radical scavenging activities of PCP and PCP-1. (**B**) ABTS radical scavenging activities of PCP and PCP-1.

As can be seen from Figure 7, PCP and PCP-1 have scavenging abilities for both DPPH and ABTS radicals. These scavenging abilities are positively correlated with sample concentration. At a sample concentration of 4 mg/mL, the clearance of DPPH by PCP and PCP-1 was 42.9% and 31.2%, respectively; the clearance of ABTS was 75.4% and 41.7%. The order of DPPH scavenging by polysaccharides and vitamin C was as follows: vitamin C > PCP > PCP-1; the order of scavenging ability of ABTS free radicals was as follows: vitamin C > PCP > PCP-1.

### 2.8. PCP and PCP-1 Reduce ROS Accumulation in C. elegans

Reactive oxygen species (ROS) are reactive chemicals that contain oxygen radicals involved in cell growth and proliferation, development, differentiation, senescence, apoptosis, and many physiopathological processes [23]. ROS are closely linked to the aging of an organism. When there is an excess of reactive oxygen radicals, it will cause lipid peroxidation and oxidative stress, destroying the cell membranes of the organism, damaging proteins, DNA, and lipids. These changes will trigger cell apoptosis and accelerate the aging process. DCFH-DA, a general-purpose oxidative stress indicator, is cell membrane-permeable and non-fluorescent. On entry into the cell, it is hydrolyzed by cytosolic esterases to form 2′,7′-dichlorodihydrofluorescein (DCFH), which is then oxidized by intracellular ROS to form the green-fluorescent 2′,7′-dichlorofluorescein (DCF). DCF can be visualized by fluorescence microscopy [24].

**Figure 8:**
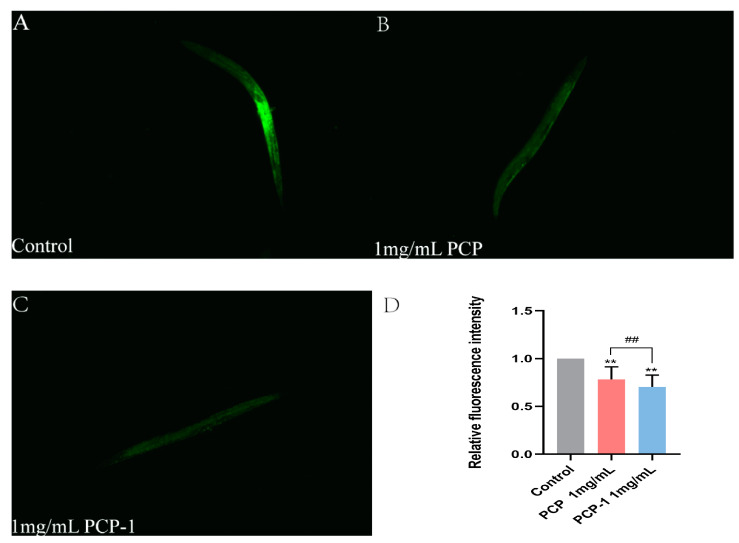
(**A**–**C**) ROS fluorescence levels in nematodes of each group. (**D**) Relative fluorescence intensity of ROS in nematodes. ** indicate the different concentrations of administered groups vs. the control group. ## indicate PCP vs. PCP-1 (1 mg/mL).

As shown in Figure 8, the level of fluorescent accumulation of ROS in nematodes was reduced in all treatment groups against the control group. Of note, when the concentration of PCP-1 was 1 mg/mL, the ROS levels were 29.66% lower than the control. The evidence was clear that polysaccharides from *Polygonatum cyrtonema* Hua reduced the accumulation of ROS in the nematode, which in turn delayed the degree of nematode senescence.

### 2.9. PCP and PCP-1 Can Induce the Nuclear Localization of DAF-16::GFP

DAF-16, a homologue of mammalian FOXO, plays an important role in the regulation of aging and resistance to stress. When the IIS pathway is inhibited, the transcription factor DAF-16/FOXO is dephosphorylated, which promotes the translocation of DAF-16 from the cytoplasm to the nucleus. Thus, it plays a role in regulating lifespan and delaying aging [25]. In order to confirm whether polysaccharides mediate the anti-aging effect by affecting the nuclear translocation of DAF-16, the transgenic nematode strain TJ356 (green fluorescent protein labeled with DAF-16) was used to observe the nuclear translocation of DAF-16. The localization of DAF-16::GFP fluorescence in nematode cells is classified into three distribution types: cytoplasmic, intermediate state, and nucleus [26]. Under normal culture conditions, DAF-16 was mainly distributed in the cytoplasm. Upon activation, DAF-16 was transferred from the cytoplasm to the nucleus, and green fluorescent dots could be observed.

**Figure 9:**
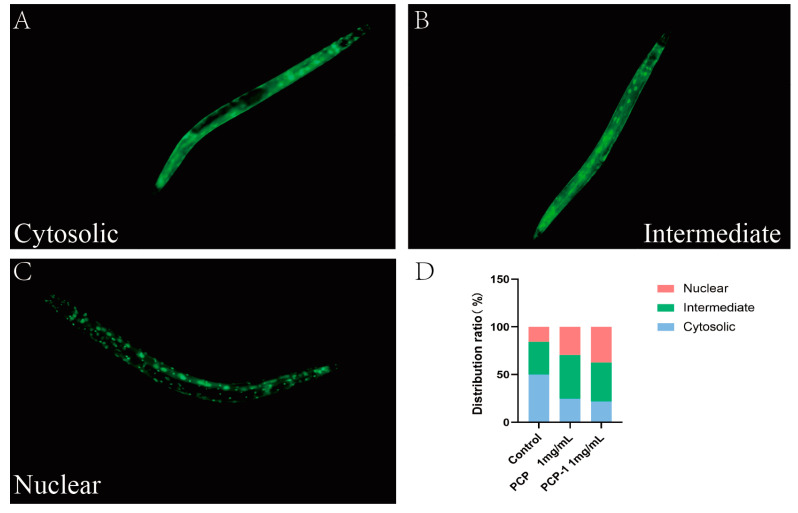
(**A**–**C**) Types of intracellular distribution of DAF-16. (**D**) Intracellular distribution of DAF-16 in different dosing groups.

As displayed in the below Figure 9, the nucleation translocation of DAF-16 appeared to be increasing in both the PCP and PCP-1 groups. This indicates that PCP and PCP-1 can induce DAF-16 nucleation and promote the accumulation of DAF-16 nucleation. In this way, they promote the accumulation of DAF-16 into the nucleus, which in turn exerts a role in delaying aging.

### 2.10. Effect of PCP and PCP-1 on Antioxidant Enzyme Activities and MDA Levels in C. elegans

**Figure 10:**
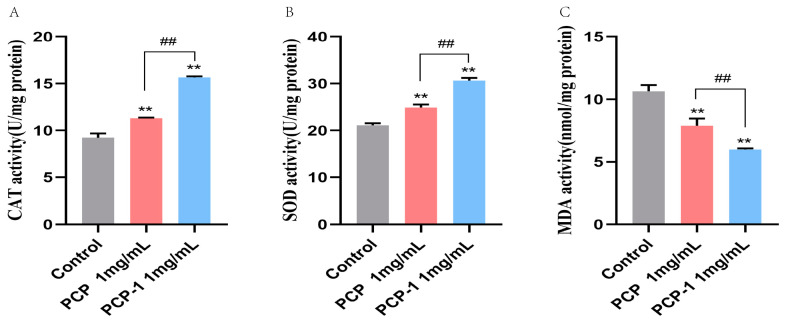
(**A**) Effect of drugs on ROS activity in *C. elegans*. (**B**) Effect of drugs on SOD activity in *C. elegans*. (**C**) Effect of drugs on MDA content in *C. elegans*. ** indicate the different concentrations of administered groups vs. the control group. ## indicate PCP vs. PCP-1 (1 mg/mL).

As we grow older, the balance of free radicals in the body gradually becomes out of kilter. Excessive superoxide radicals are generated within the body, which in turn trigger lipid peroxidation, leading to oxidative stress damage and accelerating the aging process. Antioxidant enzymes can convert superoxide radicals in the body into less toxic or harmless substances to protect cells from superoxide radical injury. For example, superoxide dismutase (SOD) catalyzes the disproportionation of superoxide anion radicals to H_2_O_2_ and oxygen. Catalase (CAT) catalyzes the decomposition of H_2_O_2_ into oxygen and water [27]. As a product of the peroxidation of free radicals with lipids, MDA is a biomarker of oxidative stress. The level of MDA in the body can reflect the degree of imbalance of oxidative stress, so MDA is also one of the indicators of the degree of human aging [28]. In two polysaccharide treatment groups(Figure 10), SOD and CAT activities significantly increased compared with those of the control group (*p* < 0.01). In the *C. elegans* pretreated with PCP and PCP-1, SOD activities were significantly increased by 17.93 and 45.17%, respectively (*p* < 0.01). Meanwhile, the activities of CAT in two treatment groups of worms increased by 22.45 and 65.52% versus untreated worms (*p* < 0.01). On the other side, the MDA levels of the treatment groups significantly decreased (Figure 10) by 25.92 and 43.71% compared with the control group, respectively (*p* < 0.01). The results revealed that PCP and PCP-1 could alleviate oxidative damage in nematodes by increasing their antioxidant capacity, thereby delaying aging. Notably, among the two polysaccharides, PCP-1 was more capable of enhancing antioxidant enzymes.

### 2.11. Effect of PCP and PCP-1 on the Expression Levels of Genes Related to the IIS Pathway in C. elegans

The IIS pathway is a classical pathway closely associated with aging. It is present in a variety of organisms, such as nematodes, drosophila, and humans. In order to investigate whether polysaccharides from *Polygonatum cyrtonema* Hua play an aging-delaying role through the IIS pathway, the expression levels of genes related to the IIS pathway in nematodes were examined using RT-qPCR technology.

**Figure 11:**
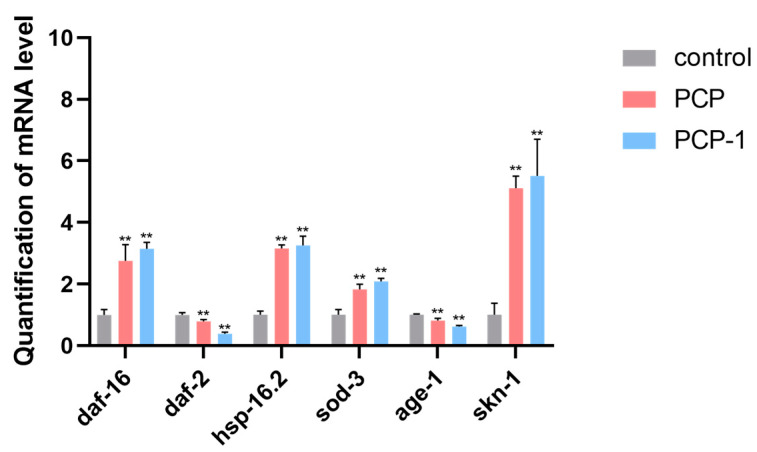
Influence of PCP and PCP-1 on the mRNA expression of *daf-16*, *daf-2*, *hsp-16.2*, *sod-3*, *age-1*, and *skn-1* in *C. elegans* N2. ** indicate the different concentrations of administered groups vs. the control group.

The results are presented in Figure 11. After 5 days of PCP and PCP-1 administration, the mRNA content of *age-1* and *daf-2* genes in the nematodes of both the PCP and PCP-1 groups showed a decreasing trend compared with the control group (*p* < 0.01). Meanwhile, the mRNA content of the *daf-16*, *skn-1*, *sod-3*, and *hsp-16.2* genes were significantly up-regulated (*p* < 0.01). This indicated that PCP and PCP-1 may exert their anti-aging effects by inhibiting IIS pathway activity in nematodes by reducing the expression levels of *daf-2* and *age-1* and increasing the expression levels of *daf-16*, *skn-1*, the heat stress-inducible gene *hsp-16.2*, and the detoxification gene *sod-3*.

## 3. Discussion

Currently, academics believe that one of the causes of organism aging is the imbalance of free radicals. When subjected to external stimulation, the body will produce excessive free radicals. When the redox balance is disrupted, excess free radicals can cause damage to the organism and trigger aging [29]. In this test, a free radical scavenging assay was used to demonstrate that PCP and PCP-1 have prominent antioxidant capacity in vitro. We noticed that the free radical scavenging rate rose with increasing concentrations of PCP and PCP-1 in the concentration range of 0.1–4.0 mg/mL. This indicated that both have a degree of antioxidant activity in vitro. However, it is of concern that PCP has a higher in vitro antioxidant capacity than PCP-1. The reason for this may be that the total polysaccharide PCP contains a portion of acidic polysaccharides. Acidic polysaccharides contain groups such as COOH and OH [30,31], which in turn enhance the antioxidant capacity of polysaccharides.

Throughout this study, we compared the activities of PCP and PCP-1 by various metrics and investigated whether the mechanisms by which they exert their aging effects are different. In terms of anti-aging activity, PCP-1 demonstrated a superior anti-aging effect when applied at similar dosages. At an equivalent level of exposure to the PCP group, PCP-1 fared better concerning longevity, stress resistance, worm growth, and lipofuscin accumulation. In the indicators for which we conducted mechanistic probes, the two did not show mechanistic differences. However, PCP-1 always more effectively promotes DAF-16 nucleation, reduces ROS and MDA levels, and improves the activities of the antioxidant enzymes SOD and CAT.

One of the most critical signaling pathways controlling development and senescence in organisms is the IIS signaling pathway. This pathway exists to play a role in regulating lifespan in a variety of organisms and is highly conserved in both nematodes and humans. In the trial, we investigated the mechanism by which PCP and PCP-1 exert their anti-aging effects by modulating the IIS pathway. DAF-2 is a receptor for the IIS pathway. The IIS pathway is activated upon binding of agonist ILPs to the receptor DAF-2. DAF-2 interacts with the insulin receptor substrate IST-1, which in turn activates AGE-1/PI3K and promotes the phosphorylation of PIP2 to PIP3. The signals provided by PIP3 activate a downstream cascade of kinases, including PDK-1, AKT-1, AKT-2, and SGK-1. These kinases promote transcription factor DAF-16/FOXO phosphorylation, prevent DAF-16 translocation to the nucleus, and inactivate it [32]. *Daf-2* and *age-1* are two key genes that function upstream of the IIS pathway. These two genes can negatively regulate *daf-16*. It was found that the genes *daf-2* and *age-1* were down-regulated in nematodes after the administration of polysaccharides from *Polygonatum cyrtonema* Hua to the nematode. This indicates that PCP and PCP-1 negatively regulate the IIS pathway by inhibiting the transcriptional activity of *daf-2*, which in turn reduces the expression level of *age-1* and then promotes the expression of *daf-16*.

DAF-16/FOXO is a vital transcription factor downstream of the IIS pathway, which can delay aging by regulating related genes downstream of the IIS pathway. In this research, the degree of the key genes downstream of the IIS pathway, *daf-16* and *skn-1*, as well as the *hsp-16.2* and *sod-3* genes regulated by them, were examined. When the IIS pathway is inhibited, it can promote the transcriptional expression of the *daf-16* and *skn-1* genes, which in turn regulates the expression of downstream stress resistance genes to exert anti-aging effects. *Hsp-16.2* is a heat stress-inducible gene [33] encoding a small HSP that enhances heat stress tolerance in nematodes and maintains protein homeostasis. *Sod-3* is an oxidative stress-inducible gene [34] encoding superoxide dismutase. It can improve nematode resilience by counteracting free radical damage to the organism. It was found that the levels of *daf-16*, *skn-1*, *hsp-16.2*, and *sod-3* were significantly increased after 5 days of medication. This means that PCP and PCP-1 regulate the expression levels of downstream-related genes *hsp-16.2* and *sod-3* through *daf-16* and *skn-1*.

In summary, we believe that the mechanism by which polysaccharides from *Polygonatum cyrtonema* Hua exert anti-aging effects may be through negative regulation of the IIS pathway and activation of the transcription factor DAF-16/FOXO to improve the stress resistance of nematodes, which in turn exerts anti-aging effects.

## 4. Materials and Methods

### 4.1. Chemicals and Reagents

*Polygonatum cyrtonema* Hua was provided by Runyuan Co. (Anhui, China) batch number 20210513. The strains used in this study were wild type N2, TJ356 (daf-16::GPF), and *E. coli* OP50. All were obtained by the Institute of Genetics and Developmental Biology, Chinese Academy of Sciences. Assay kits for BCA (batch number 20220420), MDA (batch number 20220420), SOD (batch number 20220420), and CAT (batch number 20220420) were purchased from Nanjing JianCheng Bioengineering Institute (Nanjing, China). DEAE-Sepharose Fast Flow (batch number 20210317) and DCFH-DA (batch number 1010D012) were purchased from Solarbio Co. (Beijing, China); HW-55F was purchased from Tosoh Co. (Shanghai, China), batch number 20210513; methylviologen (batch number C13950034), 5-Fluoro-2′-Deoxyuridine (batch number C12760674), and 1-Phenoxy-2-propanol (batch number M15218018) were purchased from Macklin Biochemical Technology Co. (Shanghai, China); and Trizol (batch number 1000092821) was purchased from LABLEAD Co. (Beijing, China). All other reagents were of analytical grade.

### 4.2. Extraction and Purification of PCP and PCP-1

The extraction of polysaccharides was performed as previously described [35]. The powder of *Polygonatum cyrtonema* Hua was extracted by adding 20 times the volume of water. The sample was heated and refluxed in a water bath at 80 °C two times, each time for 2 h. After combining two filtrates, they were concentrated to a specific volume under low pressure. Then, 95% ethanol was added gradually, and the mixture was stirred rapidly until the alcohol content reached 80% at 4 °C overnight. To extract the crude polysaccharide, the precipitate was filtered, repeatedly cleaned with anhydrous ethanol and acetone, and finally dried in a vacuum under reduced pressure. The purified total polysaccharide PCP was obtained by using the activated charcoal method to remove the pigment, the sevage method to remove the protein, and freeze-drying.

PCP was sequentially eluted on a DEAE-Sepharose Fast Flow chromatography column with deionized water and different concentrations of NaCl solutions. The polysaccharide fraction eluted by water was determined by further separation and purification by an HW55F gel column. Ultimately, PCP-1, a highly pure neutral polysaccharide fraction, was derived after freeze-drying.

### 4.3. UV Spectroscopy

We configured the PCP-1 (1 mg/mL) solution and scanned it at 200–400 nm. We then observed whether PCP-1 demonstrated UV absorption at 260 and 280 nm.

### 4.4. Analysis of Monosaccharide Compositions

The monosaccharide composition of PCP-1 was analyzed by the PMP pre-column derivatization HPLC method. Through comparison analysis with the retention time in the HPLC plots of each monosaccharide standard, the monosaccharide composition of PCP-1 was ascertained. For the purpose of plotting the standard curve using linear regression analysis, the mass concentration (μg/mL) of each monosaccharide standard served as the horizontal coordinate, and the corresponding peak area served as the vertical coordinate. The molar ratios of the monosaccharides in PCP-1 were calculated from the standard curve.

HPLC chromatography conditions were as follows: column: YMC-Pack ODS-A column (4.6 mm × 250 mm, 5 μm); mobile phase: 0.05 M phosphate buffer (PH = 6.8): acetonitrile (82:18); flow rate: 0.8 mL/min^−1^; injection volume: 10 μL; column temperature: 30 °C; and detection wavelength: 250 nm.

### 4.5. Anti-Aging Activities

#### 4.5.1. Exposure Experiments

A specific concentration of polysaccharides was introduced to the *E. coli* OP50 solution. These consisted of the PCP group (1 mg/mL) and the PCP-1 group (0.5, 1, and 2 mg/mL). In the control group, nematodes were cultured on NGM with *E. coli* OP50 containing distilled water added. Once spread out on NGM plates, *E. coli* OP50 fluid with various polysaccharide concentrations was allowed to dry before being used.

After using M9 buffer to wash the nematodes off the NGM plates during oviposition, they were gathered into EP tubes and centrifuged; then, the supernatant was emptied. Following the addition of lysate (H_2_O:NaClO:NaOH;8:1:1) to the EP tube, the nematodes were agitated and vortexed for 4 min. The precipitate was washed three times with M9 buffer to remove residual lysate. Precipitated eggs were transferred to NGM plates coated with *E. coli* OP50 and placed in an incubator at 22 °C for about 48 h to reach the L4 stage, which was used for subsequent experiments.

#### 4.5.2. Lifespan Assay

Pentafluorouracil at a concentration of 300 µM was also added to the NGM medium to inhibit nematode spawning. Nematodes from the L4 stage were transferred to NGM plates coated with *E. coli* OP50 bacterial solution (with or without PCP-1). One plate per group, with at least 90 worms per plate, was recorded at this point as 0 d of the longevity experiment. Death, loss, and survival of nematodes were observed and recorded at 24 h intervals, and the surviving nematodes were transferred to a new medium until all nematodes died.

#### 4.5.3. Stress Assay

##### Thermal Stress

L4-stage nematodes were transferred to new NGM plates with at least 30 worms per plate. Three days after administration, the nematodes were picked out and placed on a new plate. The Petri dishes were placed in a constant-temperature incubator at 36 °C. The survival of nematodes was counted at 1 h intervals until all of them died.

##### Oxidative Stress

Three days after dosing, the nematodes were transferred to NGM plates coated with *E. coli* OP50 bacterial solution containing 70 mM paraquat. The nematodes were cultured at a stable temperature of 22 °C. Nematode survival was counted at 12 h intervals until all nematodes had died.

#### 4.5.4. Progeny and Length Assay

During the reproductive period (approximately days 1–5), N2 worms in the L4 stage were individually transferred to fresh plates every day. One day after the plate shift, the progeny number on the original plates was recorded to calculate the mean progeny produced through the consecutive period per adult worm. For the body length experiment, nematodes were prepared for three days of administration, observed under a microscope, and photographed. Nematode body length was measured using Image J software (https://imagej.nih.gov/ij/).

#### 4.5.5. Measurement of Lipofuscin Accumulation

After being exposed to polysaccharides for 5 days, 30 nematodes in each group were randomly selected and photographed after purple fluorescence excitation with a fluorescence microscope at excitation/emission wavelengths of 400 and 430 nm. The content of lipofuscin was measured using Image J.

#### 4.5.6. In Vitro Antioxidant Assay

The antioxidant capacity of PCP and PCP-1 was determined by the DPPH and ABTS radical scavenging methods. PCP and PCP-1 were dissolved in deionized water to form final solution concentrations of 0.1, 0.2, 0.5, 1.0, 2.0, and 4.0 mg/mL. The ABTS working solution consisted of an aqueous solution of ABTS reacted with potassium persulfate at room temperature in the dark for 16 h and then diluted with anhydrous ethanol to a suitable concentration for use. The samples were added to the ABTS working solution and incubated for 6 min at room temperature. The absorbance was measured at 734 nm.

The appropriate amount of DPPH was dissolved in anhydrous ethanol and configured as a 0.2 mM DPPH working solution. The samples were added to the DPPH working solution and incubated at room temperature for 30 min. The absorbance of the samples was measured at 517 nm. As a positive control, the same concentration gradient of vitamin C was used. Distilled water was applied as a control group. The ability to scavenge activity was calculated by the following equation:

(1) $$\mathrm{scavenging}\;\mathrm{activity}\;(\%)=(1-\frac{A_{s}-A_{1}}{A_{c}})\times 100$$

Here, A_s_: represents the absorbance of the radical solution with tested polysaccharides, A_c_ represents the absorbance of the radical solution without polysaccharides, and A_1_ represents the absorbance of the distilled water with tested polysaccharides.

### 4.6. Anti-Aging Mechanisms

#### 4.6.1. Exposure Experiments

In the activity study, we compared the activity of three different concentrations of PCP-1 on the basis of several indicators of senescence. The trial findings showed that the activity indicators were more prominent when the concentration of PCP-1 was 1 mg/mL. Consequently, the administration concentration of PCP-1 was set at 1 mg/mL in the subsequent mechanistic study. The other remaining operations are the same as in Section 4.5.1.

#### 4.6.2. In Situ Measurement of Intracellular ROS Generation

Five days after administration, the cultured nematodes were collected and washed three times with M9 buffer to remove residual *E. coli* 50. We added 100 μM DCFH-DA to the mixture and allowed it to stand for 2 h at room temperature in the absence of light. After incubation and extensive washing with M9 buffer, anesthetized nematodes were viewed under a fluorescence microscope at excitation/emission wavelengths of 485 and 520 nm. The fluorescence intensity of ROS was measured with the aid of Image J.

#### 4.6.3. Nuclear Localization of DAF-16

TJ356 nematodes after five days of administration were collected in EP tubes and washed three times with M9 buffer. Anesthetized nematodes were placed under a fluorescence microscope (excitation wavelength 485 nm, emission wavelength 520 nm). the intracellular distribution of DAF-16 was analyzed with the help of Image J software.

#### 4.6.4. Kit Assay

After 5 days of L4 stage nematode administration, they were collected and rinsed three times with M9 buffer to remove residual *E. coli* OP50. The collected samples were used for the subsequent determination of antioxidant enzymes and MDA content. SOD activity, CAT activity, and MDA levels were measured by the instructions on the kits.

#### 4.6.5. RT-PCR Assay

L4-stage nematodes were administered at a constant temperature of 22 °C to about 2000 adults per group. After 5 days, the nematodes were rinsed and collected with M9 buffer. An RNA extraction reagent (Trizol) was used for sample RNA extraction. The concentration and purity of RNA were detected using an ultra-micro UV spectrophotometer. The primer sequences for the relevant genes are shown in the Table 2 below.

### 4.7. Statistical Analyses

All the tests were carried out in parallel three times. Data were analyzed and processed using GraphPad Prism 8.0 (GraphPad Software, San Diego, CA, USA). Significant comparisons were made using a one-way ANOVA. * and # represents *p* < 0.05, indicating significant differences. ** and ## represent *p* < 0.01, indicating highly significant differences.

## 5. Conclusions

In terms of chemical composition, the monosaccharide fraction of PCP-1 isolated from PCP was mannose, glucose, galactose, and arabinose (1:2.67:0.25:0.089). In examine of anti-aging, the laboratory data show that after having received PCP and PCP-1, nematode lifespan was significantly prolonged, heat resistance was increased, oxidative stress was improved, the accumulation of lipofuscin was reduced, the content of ROS and MDA in the body was lowered, the activities of antioxidant enzymes SOD and CAT were increased, and the nuclear translocation of DAF-16 was promoted.

Meanwhile, the mRNA expressed of the *age-1* and *daf-2* genes in the nematode had down-regulated and up-regulated mRNA levels of the *daf-16*, *skn-1*, *sod-3*, and *hsp-16.2* genes. Furthermore, the above analysis also revealed that PCP-1 is more advantageous in exerting anti-aging effects. The investigation implies that the mechanism by which PCP and PCP-1 exert anti-aging effects may be through negative regulation of the IIS pathway and activation of the transcription factor DAF-16/FOXO to improve the oxidative defense and stress resistance of nematodes. The discoveries herein provide some ideas and data for the development of polysaccharides from *Polygonatum cyrtonema* Hua as an antioxidant and anti-aging product.

## Figures and Tables

**Table 1:** Lifespan of nematodes in each drug group.

Sample	Average Lifespan/d	Life Extension Rate/%
Control	15.13 ± 4.06	-
PCP (1 mg/mL)	16.92 ± 3.98	11.83
PCP-1 (0.5 mg/mL)	16.98 ± 4.07	12.23
PCP-1 (1 mg/mL)	18.18 ± 3.97	20.16
PCP-1 (2 mg/mL)	17.18 ± 3.99	13.55

**Table 2:** Primer sequences for the genes of interest.

Gene	Primer Sequence (5′-3′)
*daf-16-F*	GGAGCCAAGAAGAGGATAAAGG
*daf-16-R*	GGAGAAACACGAGACGACGAT
*daf-2-F*	CGACTGAAGTGAATGGTGGA
*daf-2-R*	CGCCGTTACTGAGACAAAATA
*age-1-F*	CGCTGGCATCAAAATCTACA
*age-1-R*	ATTGGCAGTCGGTTCAGGAG
*hsp-16.2-F*	CGCTATCAATCCAAGGAGAAC
*hsp-16.2-R*	GAAGCAACTGCACCAACATC
*sod-3-F*	AGCATCATGCCACCTACGTGA
*sod-3-R*	CACCACCATTGAATTTCAGCG
*skn-1-F*	TAGCCGACGACGAAGAAGAG
*skn-1-R*	AGGTGTTGGACGATGGTGAA

## Data Availability

Data are contained within the article.
